# Interrelationships between *ALOX5AP* Polymorphisms, Serum Leukotriene B4 Level and Risk of Acute Coronary Syndrome

**DOI:** 10.1371/journal.pone.0106596

**Published:** 2014-09-11

**Authors:** Guoping He, Shan Ye, Jingjiao Hui, Dandan Shen, Chuanping Qi, Lianhong Xu, Yichao Qian

**Affiliations:** 1 Department of Cardiology, Affiliated Wujin Hospital of Jiangsu University, Changzhou, Jiangsu, China; 2 Central Laboratory, Affiliated Wujin Hospital of Jiangsu University, Changzhou, Jiangsu, China; National Cheng Kung University Medical College, Taiwan

## Abstract

**Background:**

We investigated the relationships between the *ALOX5AP* gene rs10507391 and rs4769874 polymorphisms, serum levels of leukotriene (LT) B4, and risk of acute coronary syndrome (ACS).

**Methods:**

A total of 709 participants, comprising 508 ACS patients (ACS group) and 201 noncoronary artery disease patients with chest pain (control group) were recruited from the Han population of the Changwu region in China. Two polymorphic loci were genotyped using polymerase chain reaction and restriction fragment length polymorphism analysis. Serum LTB4 level was determined by enzyme-linked immunosorbent assay.

**Results:**

Serum LTB4 levels were significantly higher (*P*<0.001) in the ACS group (median/interquartile range, 470.27/316.32 pg/ml) than in the control group (233.05/226.82 pg/ml). No statistical differences were observed between genotype, allele and haplotype frequencies for the tested loci in either the ACS group or the control group, even after adjustments were made for conventional risk factors by multivariate logistic regression. This suggests there is no association between the *ALOX5AP* rs10507391 and rs4769874 polymorphisms and ACS risk. Elevated serum LTB4 level was closely linked to ACS risk, and may be independent of traditional risk factors as a risk factor for ACS (*P*<0.001). There was no significant association between serum LTB4 levels and the two variants in either the ACS group or the control group.

**Conclusions:**

Rs10507391, rs4769874 and its haplotypes in *ALOX5AP* are unrelated to ACS risk in the Chinese Han population of Changwu, but elevated serum LTB4 level is strongly associated with ACS risk. Serum LTB4 level is not subject to the influence of either the rs10507391, rs4769874 or the haplotype.

## Introduction

Acute coronary syndrome (ACS) frequently occurs in atherosclerotic patients with myocardial ischemia. ACS, which has high mortality and disability rates, is often triggered when plaques rupture or fracture, leading to thrombosis. Leukotrienes (LTs) are inflammatory mediators derived from arachidonic acid via the 5-lipoxygenase pathway, and experimental and clinical studies implicate the 5-lipoxygenase pathway in the pathophysiology of atherosclerosis [1]. In particular, leukotriene B4 (LTB4) is a chemoattractant that promotes leukocyte adhesion and diapedesis through the endothelial cell barrier, and also induces chemotaxis and cell proliferation in the human coronary artery [2]. Variations in the *ALOX5AP* gene, which encodes arachidonate 5-lipoxygenase-activating protein, reportedly conferred an increased risk of myocardial infarction and stroke, independent of conventional risk factors [3], [4]. However, these results proved difficult to replicate. Two studies in European populations and a meta-analysis found *ALOX5AP* associations with stroke, myocardial infarction, and coronary artery disease (CAD) [5]–[7], but two separate studies and an additional meta-analysis did not [8]–[10]. In addition, as early as 2004, Helgadottir et al. found that LTB4 production from calcium-ionophore-stimulated blood neutrophils was more prevalent in myocardial infarction cases than in healthy controls [3]. In this study, we aim to explore the interrelationship between *ALOX5AP* gene variants rs10507391 and rs4769874, serum LTB4 level, and ACS risk, in a Chinese Han population from the Changwu region.

## Materials and Methods

### Study population

The study was approved by Changzhou Wujin People' Hospital ethics committee. All participants enrolled gave written informed consent. A total of 709 unrelated, ethnically matched, consecutive individuals from the Han population of the Changwu region of China were recruited to the study. These individuals, who were admitted to our hospital from July 2006 to July 2011, consisted of 508 patients with ACS (ACS group; 369 male and 139 female) and 201 cases of nonCAD patients with chest pain (control group; 105 male and 96 female). The ACS group included 329 cases of acute myocardial infarction (247 male and 82 female) and 179 cases of unstable angina pectoris (122 male and 57 female). ACS was diagnosed according to the 2002 criteria of the American College of Cardiology (ACC)/American Heart Association (AHA) [11]. NonCAD patients were categorized using clinical history, physical examination, electrocardiography, exercise tests, and coronary angiography (without coronary stenosis). Patients with cardiomyopathy, tumor, renal or hepatic insufficiency, rheumatism, or severe infection (acute or chronic) were excluded from the study. Smoking history (ever having smoked) was ascertained by interview. The study population characteristics are shown in Table 1. Hypertension was defined by systolic blood pressure ≥140 mmHg and/or diastolic blood pressure ≥90 mmHg and/or taking antihypertensive drugs. Diabetes mellitus (DM) was defined by blood glucose ≥126 mg/dL (7.0 mmol/L) and/or taking hypoglycemic medication. Hyperlipidemia was defined by total cholesterol ≥240 mg/dL (6.19 mmol/L), low-density lipoprotein cholesterol ≥160 mg/dL (4.14 mmol/L), triglycerides ≥200 mg/dL (2.27 mmol/L) and/or taking antihyperlipidemic agents.

**Table 1:** Characteristics of the ACS and control groups.

	Controls (n = 201)	ACS (n = 508)	P-value
Male	105 (52.24%)	369 (72.64%)	**<0.001**
Hypertension	74 (36.82%)	276 (54.33%)	**<0.001**
Smokers	24 (11.94%)	140 (27.56%)	**<0.001**
Diabetes mellitus	11 (5.47%)	104 (20.47%)	**<0.001**
Age, years	57.92±9.56	63.73±10.67	**<0.001**
Total cholesterol (mmol/L)	4.52±0.98	4.60±1.00	0.394
Triglyceride (mmol/L)	1.76±1.11	1.82±1.34	0.634
High-density lipoprotein cholesterol(mmol/L)	1.20±0.43	1.28±3.90	0.761
Low-density lipoprotein cholesterol(mmol/L)	2.58±0.74	2.77±0.80	**0.005**

ACS: Acute coronary syndrome

### Detection of rs10507391 and rs4769874 polymorphisms in the *ALOX5AP* gene

Rs10507391 and rs4769874 polymorphisms in the *ALOX5AP* gene were investigated. Genomic DNA was extracted from peripheral blood leukocytes using a standard phenol-chloroform method. Primers used for detecting polymorphisms were synthesized by Sangon Biotech (Shanghai) and were as follows: 1) rs10507391 (forward) 5′ GTG TTC AGG AAG GGA GTT TCT GT 3′ and (reverse) 5′ GTC TAT GGT TGC AAC ATT GAG ATT A 3′; and 2) rs4769874 (forward) 5′ CCC ACT TTC CTC GCT GTG CT 3′ and (reverse) 5′ CCG AAA GGG GAC CAA AAG TA 3′. Polymerase chain reaction (PCR) was performed in a 25 µl reaction volume containing 1 µl (0.1 µg) of genomic DNA, 12.5 µl of Premix Ex Taq DNA polymerase (Takara Biotechnology, Dalian), 1 ul of each primer and 9.5 µl of sterile water. PCR conditions were as follows: 95°C for 5 min; (95°C for 30 s; 60°C for 30 s; 72°C for 40 s) ×30 cycles; 72°C for 10 min. PCR products were subsequently restriction-digested at 37°C overnight for restriction fragment length polymorphism (RFLP) analysis. rs10507391 products were digested with VspI (Takara Biotechnology, Dalian), and rs4769874 products were digested with BstuI (New England Biolabs). The digested products were electrophoresed on 3% (rs10507391) and 2% (rs4769874) agarose gels and genotypes were determined using a gel imaging and analysis system. Several PCR products were sequenced to verify RFLP data.

### Quantification of LTB4 level

Serum LTB4 level was quantified using an enzyme-linked immunosorbent assay (ELISA) kit (Adlitteram Diagnostic Laboratories).

### Statistical analysis

Serum LTB4 levels (skewed distribution values) and enumeration data are expressed as a median/interquartile range (M/IQR) and percentage (%), respectively. Other data are expressed as mean with standard deviation. Serum LTB4 levels were compared between binomial genotypes using a Kruskal-Wallis test. Differences between groups were tested using independent student's t-tests. Two-sample t-tests for independent samples were used to compare other measurement data across groups. Chi-square tests were used to determine genotype distributions for Hardy-Weinberg equilibrium and to compare allele and genotype frequencies between patients and controls. Multiple logistic regression analysis was performed to explore the effects of polymorphisms and serum LTB4 levels on ACS risk, and are presented as odds ratios (OR) with 95% confidence intervals (95% CI). Above-mentioned analyses were performed using the SPSS 16.0 software package. Haplotype frequencies were estimated using SHEsis (http://analysis.bio-x.cn/myAnalysis.php). A P-value of <0.05 was considered statistically significant.

## Results

### Clinical characteristics

Baseline characteristics for all participants are shown in Table 1. Median age, proportion of males, number of smokers, levels of hypertension, incidence of DM, and serum low-density lipoprotein cholesterol level were higher in the ACS group than in the control group. No significant differences between the groups were detected with respect to total serum cholesterol, triglyceride, or high-density lipoprotein cholesterol levels.

### Rs10507391 and rs4769874 polymorphisms in the *ALOX5AP* gene

**Figure 1:**
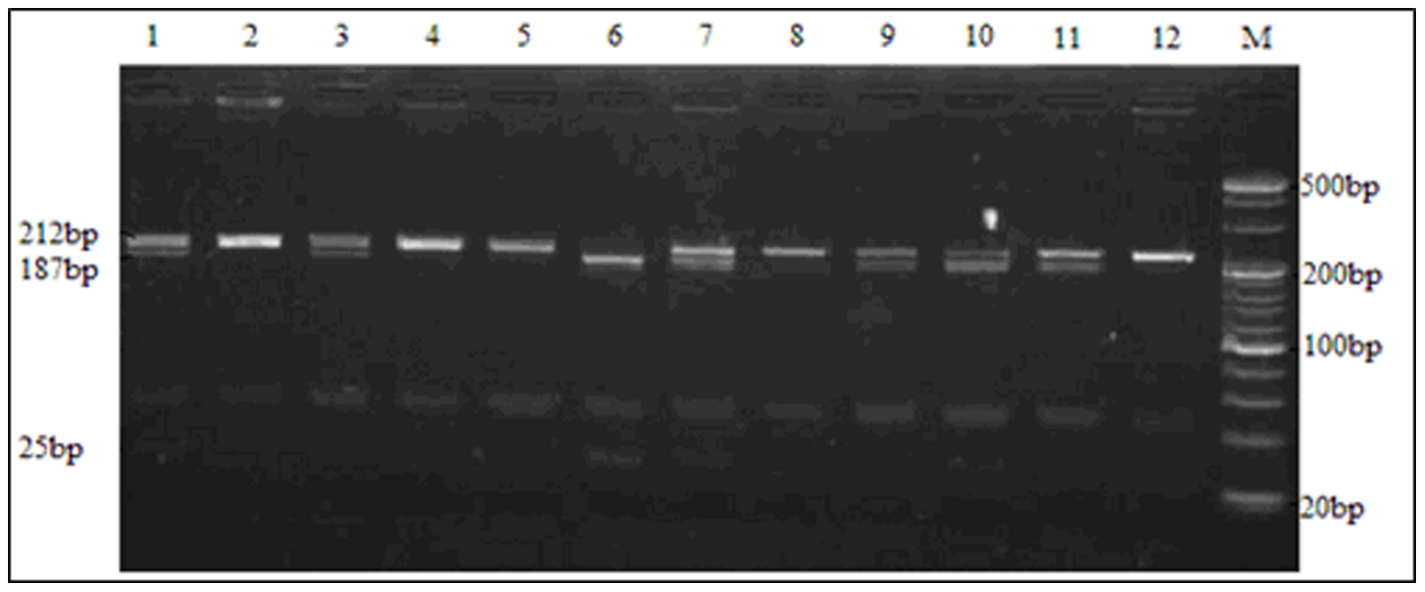
Determination of the rs10507391 genotype by PCR amplification and restriction analysis. When the nucleotide A is present, a Vsp I restriction site is created. Lane 6: homozygous AA (187 bp and 25 bp). Lane 1,3,7,9–11: heterozygous AT (212 bp,187 bp and 25 bp). Lane 2,4,5,8,12: homozygous TT (212 bp). Lane M: DNA marker.

**Figure 2:**
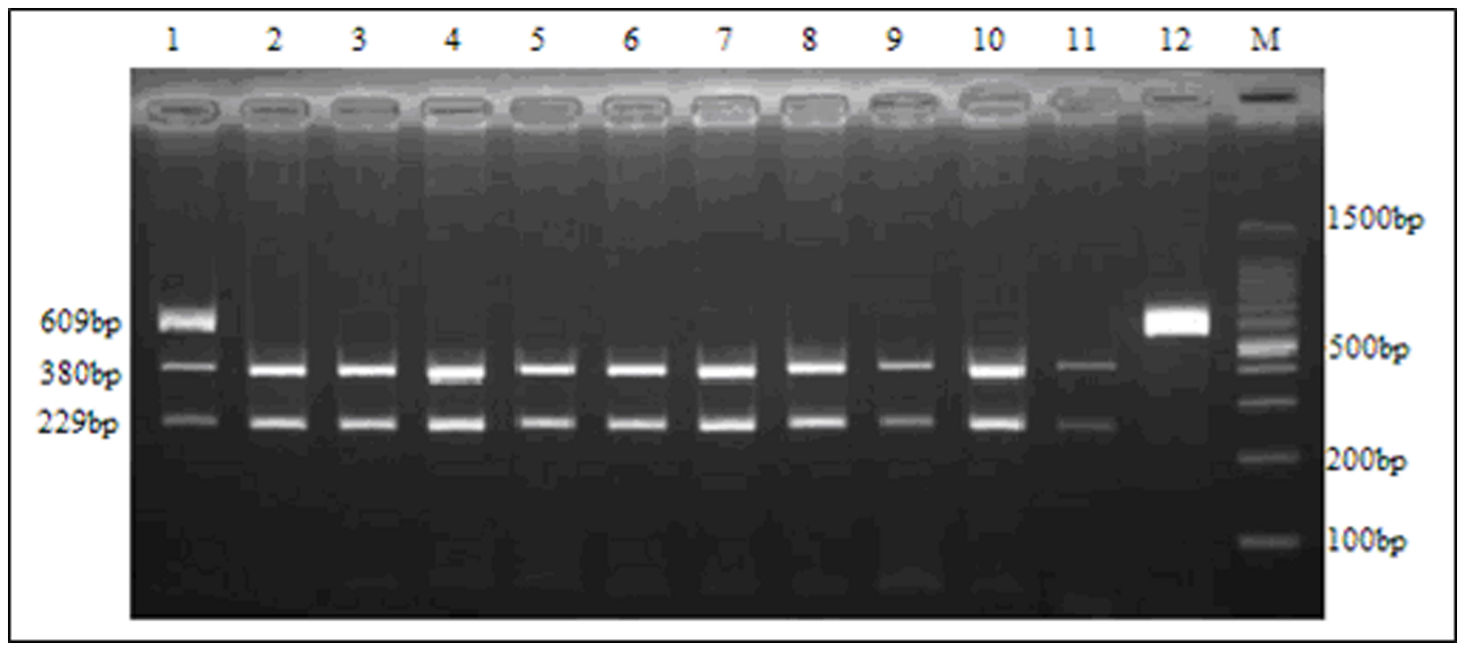
Determination of the rs4769874 genotype by PCR amplification and restriction analysis. When the nucleotide G is present, an Bstu I restriction site is created. Lane 2–11: homozygous GG (380 bp and 229 bp). Lane1: heterozygous GA (609 bp,380 bp and 229 bp). Lane 12: homozygous AA (609 bp). Lane M: DNA marker.

**Figure 3:**
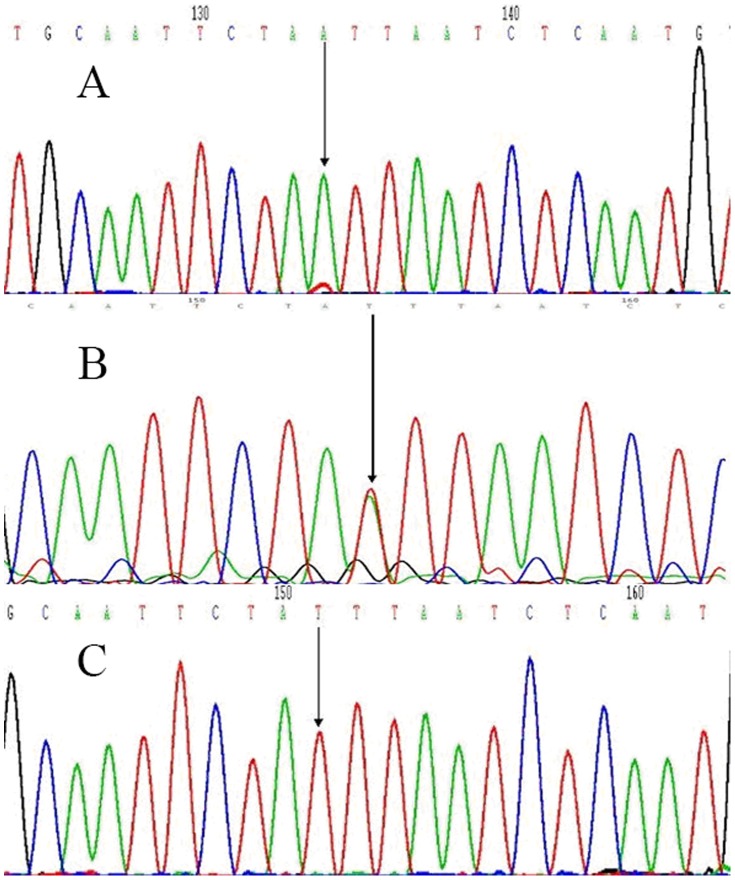
Sequenced diagrams of the PCR amplification production of the rs10507391. (A) A unimodal arrowed was homozygous AA. (B) A/T bimodal arrowed was heterozygous AT. (C) T unimodal arrowed was homozygous TT.

**Figure 4:**
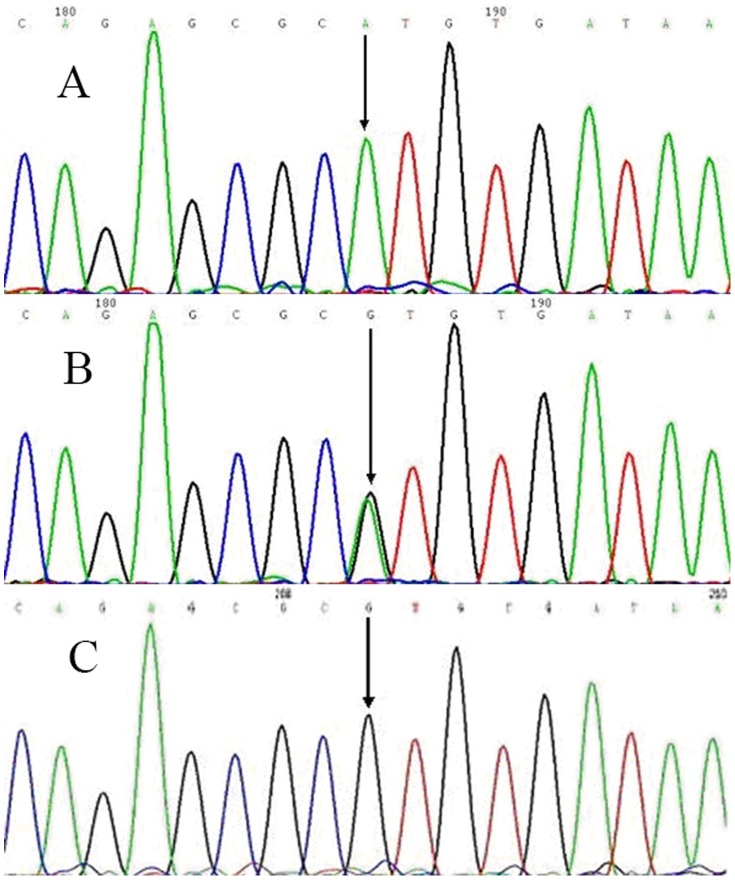
Sequenced diagrams of the PCR amplification production of the rs4769874. (A) A unimodal arrowed was homozygous AA. (B) GA bimodal arrowed was heterozygous GA. (C) G unimodal arrowed was homozygous GG.

For rs10507391, three genotypes were distinguished after digestion of the 212 bp PCR product: homozygous AA (187 bp, 25 bp), heterozygous AT (212 bp, 187 bp, 25 bp), and homozygous TT (212 bp) (Fig. 1). Three genotypes were also distinguished through digestion of the 609 bp rs4769874 product: homozygous GG (380 bp, 229 bp), heterozygous GA (609 bp, 380 bp, 229 bp) and homozygous AA (609 bp) (Fig. 2). Diagrams of the sequenced PCR amplicons are shown in Figures 3 and 4. Chi-square analysis indicated that these data are consistent with Hardy-Weinberg genetic equilibriums (P-value >0.05) (Table 2).

**Table 2:** Hardy-Weinberg genetic equilibriums for *ALOX5AP* genotype frequency in control and ACS groups.

	Genotype			Allele		χ^2^	P-value
**rs10507391**	**AA**	**AT**	**TT**	**A**	**T**		
Controls (n = 201)	18 (8.96)	102 (50.75)	81 (40.30)	138 (34.33)	264 (65.67)	3.17	0.075
ACS (n = 508)	53 (10.43)	247 (48.62)	208 (40.94)	353 (34.74)	663 (65.26)	2.65	10.34
**rs4769874**	**GG**	**GA**	**AA**	**G**	**A**		
Controls (n = 201)	189 (94.03)	11 (5.47)	1 (0.50)	389 (96.77)	13 (3.23)	3.17	0.075
ACS (n = 508)	486 (95.67)	21 (4.13)	1 (0.20)	993 (97.74)	23 (2.26)	2.20	0.138

ACS: Acute coronary syndrome.

Data are provided as number of individuals and (percentage).

### Rs10507391 and rs4769874 polymorphisms are not related to ACS risk

All possible heterozygote and homozygote combinations were detected for the rs10507391 and rs4769874 *ALOX5AP* polymorphisms in the study population of 709 individuals. Genotype and allele prevalence did not differ significantly between the ACS and the control groups, even after adjustments for conventional risk factors by multivariate logistic regression (P-values >0.05) (Table 3).

**Table 3:** Distribution of *ALOX5AP* polymorphisms in ACS and control groups.

	Controls	ACS	Odds ratio (95% CI)	P-value	# Odds ratio (95% CI)	P-value
**rs10507391**						
Genotype						
AA	18 (8.96)	53 (10.43)	0.844 (0.482–1.481)	0.583	1.143 (0.620–2.107)	0.669
AT	102 (50.75)	247 (48.62)	1.089 (0.785–1.509)	0.618	0.870 (0.610–1.240)	0.441
TT	81 (40.30)	208 (40.94)	0.974 (0.698–1.358)	0.932	1.102 (0.768–1.580)	0.599
Allele						
A	138 (34.33)	353 (34.74)	0.982 (0.770–1.252)	0.902	1.030 (0.781–1.358)	0.835
T	264 (65.67)	663 (65.26)				
**rs4769874**						
Genotype						
GG	189 (94.03)	486 (95.67)	0.439 (0.429–0.448)	0.357	1.662 (0.759–3.639)	0.204
GA	11 (5.47)	21 (4.13)	0.545 (0.536–0.555)	0.439	0.617 (0.274–1.388)	0.243
AA	1 (0.50)	1 (0.20)	1.000 (1.000–1.000)	0.496	0.454 (0.024–8.534)	0.598
Allele						
G	389 (96.77)	993 (97.74)	0.342 (0.332–0.351)	0.295	1.596 (0.780–3.263)	0.200
A	13 (3.23)	23 (2.26)				

ACS: Acute coronary syndrome.

Data are shown as number of individuals and (percentage).

# Odds ratios adjusted for conventional risk factors including gender, age, history of smoking, hypertension, diabetes mellitus, and dyslipidemia.

### Haplotype analysis of the two variants

We calculated the Linkage Disequilibrium test of the rs10507391 and rs4769874 by SHEsis showed that D′ = 0.246 and r2 = 0.003, weak LD between the two variants was observed. Then we continued to conduct a haplotype analysis. Two haplotypes had an estimated frequency below 3% (AA and TA) were not shown, the other two (AG and TG) showed no statistical difference between the ACS and the control groups (P-values >0.05) (Table 4). This suggests that no relationships exist between ACS risk and the two polymorphisms tested.

**Table 4:** Haplotype frequencies of ALOX5AP genetic variants.

Haplotype	ACS	Control	P	OR	95%CI
AG	340.04 (0.335)	132.48 (0.330)	0.947	1.008	0.787∼1.291
TG	652.96 (0.643)	256.53 (0.638)	0.947	0.992	0.774∼1.270

ACS: Acute coronary syndrome.

### Serum LTB4 levels are higher in ACS patients than in controls

**Figure 5:**
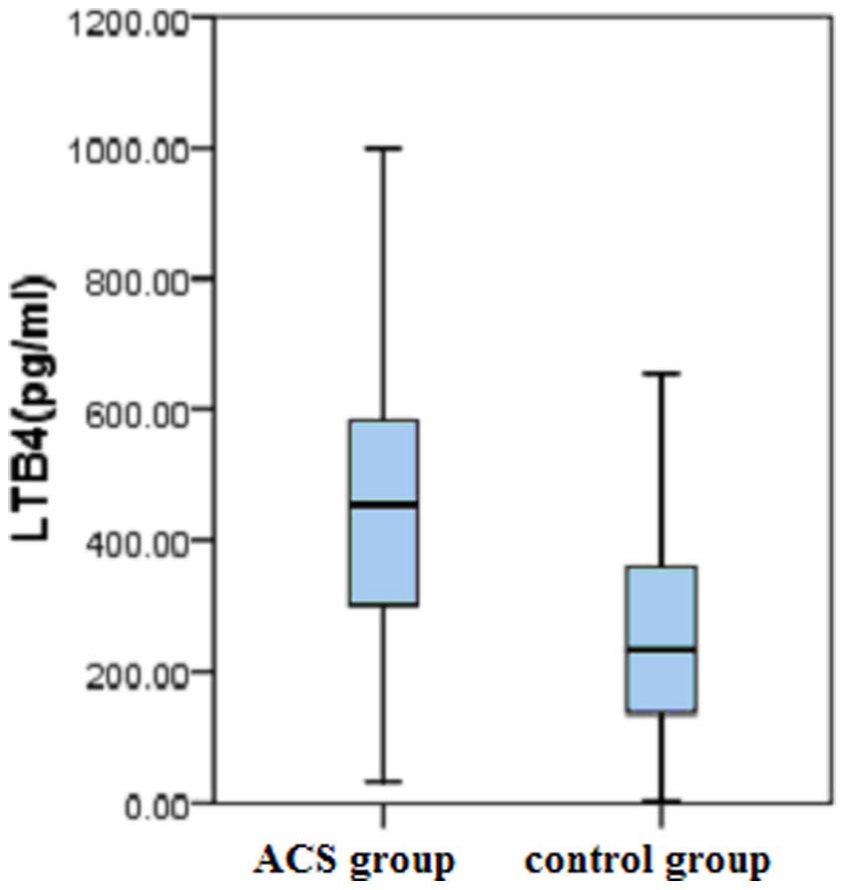
Plot box of serum LTB4 levels (pg/ml) between ACS and control groups. Serum LTB4 level in ACS patients was significantly higher than those in controls (P value is less than 0.001).

Serum LTB4 levels were significantly higher in ACS patients than in controls (M/IQR: 470.27/316.32 pg/ml vs. 233.05/226.82 pg/ml, P<0.001) (Fig. 5). Elevated serum LTB4 level was significantly associated with an increased ACS risk, after adjustment for conventional risk factors such as gender, age, history of smoking, hypertension, DM, and dyslipidemia, using multivariate logistic regression with nonCAD patients as a reference group (P<0.001).

### Serum LTB4 levels are not significantly associated with *ALOX5AP* genotype

**Figure 6:**
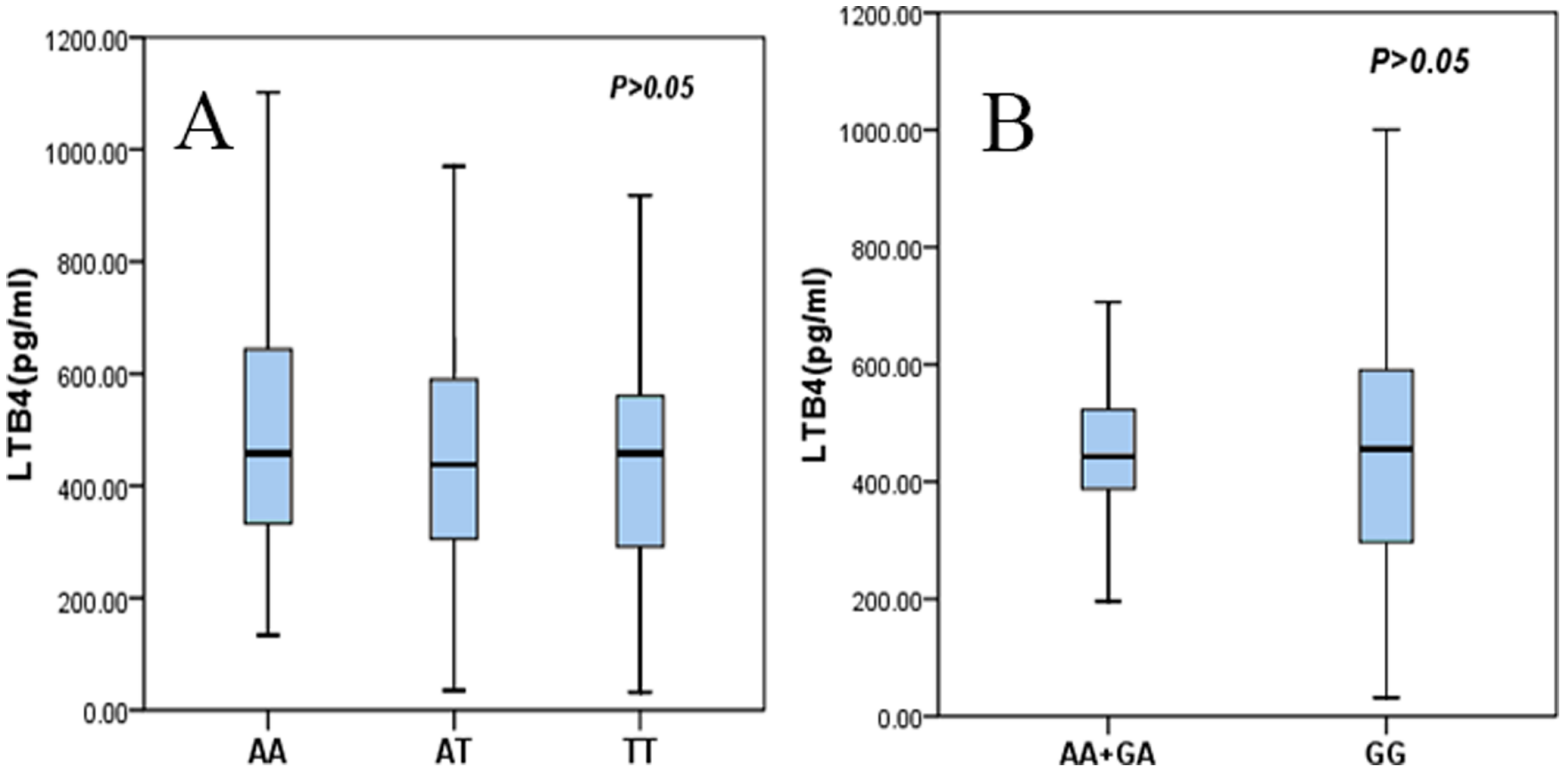
Plot box of serum LTB4 levels (pg/ml) among ALOX5AP genotypes in ACS patients. (A) Serum LTB4 level was similar among AA, AT and TT genotype of rs10507391. (B) Serum LTB4 level also was similar between (AA+GA) and GG genotype of rs4769874.

**Figure 7:**
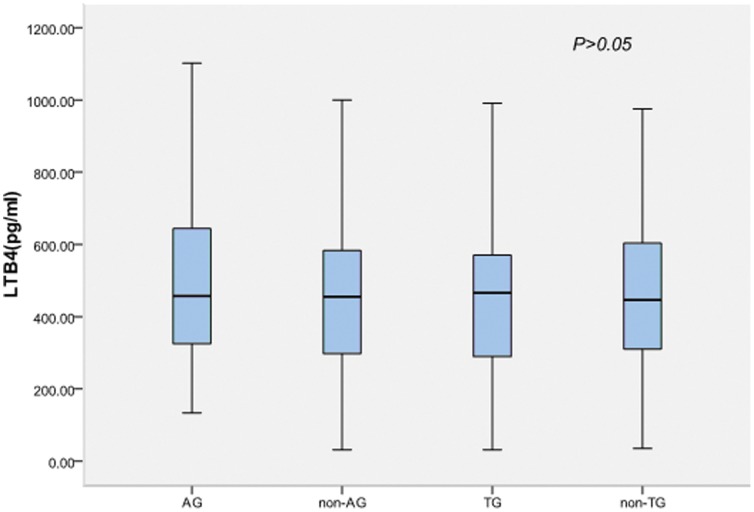
Plot box of serum LTB4 levels (pg/ml) between ALOX5AP haplotypes in ACS patients.

There were no associations between serum LTB4 level and the rs10507391, rs4769874 (even with pairwise genotype comparisons) or its haplotype (Fig. 6–7, Table 5–6). Multivariate logistic regression adjusting for conventional risk factors such as gender, age, history of smoking, hypertension, DM, and dyslipidemia revealed no association between serum LTB4 levels and the *ALOX5AP* polymorphisms in either the ACS group or the control group (Table 5–6).

**Table 5:** Serum LTB4 levels and *ALOX5AP* polymorphisms in ACS and control groups.

	Genotype (n)	LTB4 (pg/ml)	Pairwise compared	P-value	#Odds ratio (95% CI)	P-value
**rs10507391**						
ACS (n = 508)	AA (53)	458.04/325.91	AA:AT	0.699	0.994 (0.542–1.821)	0.984
	AT (247)	468.62/324.11	AT:TT	0.721	1.076 (0.741–1.561)	0.700
	TT (208)	473.22/302.95	AA:TT	0.589	1.114 (0.604–2.056)	0.729
Control (n = 201)	AA (18)	201.15/200.11	AA:AT	0.114	2.031 (0.678–6.082)	0.205
	AT (102)	233.20/257.60	AT:TT	0.533	1.181 (0.644–2.165)	0.591
	TT (81)	254.24/219.96	TT:AA	0.045	1.674 (0.537–5.218)	0.374
**rs4769874**						
ACS (n = 508)	AA+GA (22)	442.99/140.93	(AA+GA):GG	0.666	1.256 (0.530–2.978)	0.605
	GG (486)	470.78/323.43				
Control (n = 201)	AA+GA (12)	280.58/310.05	(AA+GA):GG	0.963	0.840 (0.246–2.869)	0.781
	GG (189)	233.05/225.84				

ACS: Acute coronary syndrome.

# Odds ratios adjusted for conventional risk factors including gender, age, history of smoking, hypertension, diabetes mellitus, and dyslipidemia.

LTB4 levels are expressed as median/interquartile range.

**Table 6:** Serum LTB4 levels and haplotypes in ACS and control groups.

	Haplotype (n)	LTB4 (pg/ml)	P-value	#Odds ratio (95% CI)	P-value
ACS (n = 508)	AG (50)	457.33/325.14	0.735	1.079 (0.595–1.957)	0.803
	non-AG (458)	470.78/316.12			
Control (n = 201)	AG (16)	201.15/202.162	0.066	2.399 (0.765–7.526)	0.134
	non-AG (185)	240.39/235.01			
ACS (n = 508)	TG (203)	491.31/310.40	0.771	1.178 (0.820–1.690)	0.375
	non-TG (305)	462.39/318.33			
Control (n = 201)	TG (77)	257.48/201.71	0.138	1.276 (0.701–2.321)	0.425
	non-TG (124)	223.94/245.35			

ACS: Acute coronary syndrome.

# Odds ratios adjusted for conventional risk factors including gender, age, history of smoking, hypertension, diabetes mellitus, and dyslipidemia.

LTB4 levels are expressed as median/interquartile range.

## Discussion

ACS often occurs with a background of atherosclerosis, which is attributed to the interactions of multiple genetic and environmental risk factors. Atherosclerosis is potentially fatal, and can lead to acute myocardial infarction, unstable angina pectoris and sudden cardiac death. In our study, male, elderly, hypertension, diabetes, smoking, and hypercholesterolemia were confirmed as risk factors for ACS. Nevertheless, increasing numbers of studies indicate that genetic factors can also significantly contribute to individual ACS risk, suggesting scope for future personalized disease prevention and treatment [12]–[17]. For example, the DeCode study highlighted gene polymorphisms and haplotypes associated with *ALOX5AP* [3]. One such haplotype, HapA (composed of rs17222814, rs10507391, rs4769874 and rs9551963), was linked to a doubled risk of myocardial infarction and stroke in patients from Iceland. A second haplotype, HapB (composed of rs17216473, rs10507391, rs9315050 and rs17222842), was associated with myocardial infarction in individuals from the United Kingdom [3], and was particularly overrepresented in male ischemic stroke patients in a Scottish population [4]. Our present study found no associations between ACS risk and *ALOX5AP* two variants or haplotype in a Chinese Han cohort from the Changwu region, suggesting that there is no link between *ALOX5AP* and coronary artery disease in this population.

Now it is generally deemed that inflammation promotes formation and rupture of plaque is the main pathogenic mechanism of atherosclerosis [18]. The 5-lipoxygenase pathway produces LTs, which are inflammatory mediators, from arachidonate [19], [20], and is implicated in atherosclerosis pathogenesis [21], [22]. Furthermore, LTB4 production by calcium-ionophore-stimulated blood neutrophils was found to be higher in myocardial infarction cases than in healthy controls [4]. Our data support these results; serum LTB4 level were significantly higher in ACS patients than in nonCAD patients. Elevated serum LTB4 level was closely related to ACS risk, providing further evidence for LTB4 as an important inflammatory factor in the course of catadrome and ACS progression.

So, what relation exist between serum LTB4 level and ALOX5AP gene polymorphism? The DeCode study further suggested that ALOX5AP gene is involved in the pathogenesis of CAD by increasing LTs production and inflammation in the arterial wall, leading to stenosis, damage and rupture, finally cardiovascular events [3]. Maznyczka et al. found no evidence for increased LTB4 production by blood neutrophils when stimulated with calcium ionophore A23187 in healthy subjects carrying either *ALOX5AP* HapA or HapB when compared with non-A/non-B carriers, they thought the mechanism was not simply due to a genetic effect, but what exactly, they were not very clear [23]. In our study, no significant differences in serum LTB4 level were found between any of the *ALOX5AP* rs10507391, rs4769874 or its haplotypes in either ACS or nonCAD patients. This suggests that the rs10507391, rs4769874 and its haplotypes do not influence serum LTB4 levels. ACS is a multifactor disease affected by environmental and genetic factors, and serum LTB4 level may also be affected by environmental and genetic factors. Therefore, any association between *ALOX5AP* polymorphisms and serum LTB4 levels remains to be clarified.

It may be possible to explain the seemingly mutually exclusive data arising from the different studies. In our study, we investigated the effects of only two *ALOX5AP* loci, rs10507391 and rs4769874, and its haplotypes, HapA and HapB had not been built. Currently, we cannot eliminate the possibility that other, as yet unknown, polymorphisms may be influencing LTB4 level, or that unknown environmental factors may modulate the effect of the known polymorphisms. In addition, we measured LTB4 levels in circulating blood, which may not necessarily represent LTB4 levels at biologically significant locations.

## Conclusion

In summary, we confirmed the presence of *ALOX5AP* polymorphisms at rs10507391 and rs4769874 in subjects from a Han population in the Changwu region of China. No significant difference in polymorphism incidence was found between ACS and nonCAD subjects, indicating that these polymorphisms were not linked to ACS in this population. Serum LTB4 level was higher in ACS patients than in nonCAD controls, and elevated serum LTB4 levels were related to an increased risk to ACS. Notably, we found that serum LTB4 level was unrelated to the *ALOX5AP* polymorphisms in both the ACS and the nonCAD individuals. However, our study was limited: sample size was small, study subjects were from a small geographical region, and LTB4 levels were measured only once. Additional research with larger cohorts will be needed to confirm the conclusions of this study.
